# Editorial: New Advances in Nanomaterials

**DOI:** 10.3390/ma16093535

**Published:** 2023-05-05

**Authors:** Cheng Wang, Xiu Yu, Maning Liu, Zhifeng Deng, Daohai Zhang, Haichang Zhang

**Affiliations:** 1 Key Laboratory of Rubber-Plastics of Ministry of Education/Shandong Province (QUST), School of Polymer Science and Engineering, Qingdao University of Science and Technology, Qingdao 266061, China; 2020020066@mails.qust.edu.cn (C.W.); 13355423640@163.com (X.Y.); 2 Centre for Analysis and Synthesis, Department of Chemistry, Lund University, 22100 Lund, Sweden; maning.liu@tuni.fi; 3 School of Materials Science and Engineering, Shaanxi University of Technology, Hanzhong 723001, China; dengzf@snut.edu.cn; 4 School of Chemical Engineering, Guizhou Minzu University, Guiyang 550025, China; zhangdaohai6235@163.com

In the past few years, people have been committed to a variety of properties and functional materials, among which are nanomaterials, which have been gradually developed in-depth [1,2,3,4]. Because of the small scale of the constituent unit of nanomaterials, their interfaces occupy a considerable portion of their composition. Therefore, nanomaterials have a variety of characteristics, such as mechanical properties [5], electrical properties [6], magnetic properties [7], and thermal properties [8], among others, which are different from those of other materials formed by the same chemical elements. Furthermore, the special properties of nanomaterials have been applied in various scientific and technological fields, while being accompanied by rapid development. In addition, the nanometer system has enabled a new level of understanding of the nature of nanomaterials, since they are the intermediate link between atoms, molecules, and macroscopic systems, which includes a deeper comprehension of the transition from micro to macro. This Special Issue includes ten original research works, including the application of nanostructured materials in the field of biomedicine and material processing.

In the field of biomedical health, nanomaterials have been widely used for a variety of detection and antibacterial purposes. With the acceleration of human industrialization and urbanization, the pollution of land estuarine water has become one of the most serious environmental problems, which is mainly manifested by the abnormal increase in minerals and severe eutrophication. These impacts pose serious challenges to the ecological environment and the aquaculture industry. Therefore, Zaimi Xie et al. were committed to optimizing the algorithm and proposed the PCA-EEMD-CNN-Attention-GED seawater aquaculture water quality prediction model, which changed the traditional on-site water quality monitoring method [9]. The root means square error (RMSE), mean absolute percentage error (MAPE), and decision coefficient (R2) of the obtained short-term monitoring data were 0.246%, 0.307%, and 97.80%, respectively, and the predicted RMSE, MAPE, and R2 of their long-term series were 0.878%, 0.594%, and 92.23%, respectively. The results indicate that this prediction model can more accurately monitor water quality compared to traditional prediction models and can provide a decision-making basis for water quality control and management in aquaculture.

Silver nanoparticles (Ag NPs) have drawn a great deal of attention and research in the fields of medicine and biology due to their excellent antibacterial activity. At present, the most popular method for preparing Ag NPs is chemical reduction, in which the end-capping agents are typically biologically toxic polyvinyl pyrrolidine (PVP) and bovine serum albumin (BSA). To address these issues, Xingyun Yang et al. synthesized extracellular polysaccharide (EPS)-coated Ag NPs using three parts of the EPS produced by fungi [10]. The effects of reaction time, temperature, reagent concentration and other factors on the synthesis of Ag NP were studied. The synthesis procedure in this work did not involve any organic solvents or harsh experimental conditions, which indicates that this method is a green, simple, and convenient method of synthesis.

Various nanomaterials are frequently added to cement-based and geopolymer matrices in order to improve their mechanical properties. However, the dispersibility of nanomaterials significantly impacts the performance of composite materials. A series of tests were conducted on the rheological properties of geopolymers by Madeleing Taborda Barraza’s group [11]. In this study, the influence of carbon nanotubes (CNT) and silicon carbide whiskers (SCW) with similar geometric shapes on the rheological properties of metakaolin-based geopolymers was evaluated. The studies indicated that the addition of nano materials could significantly improve the average mechanical property matrix. For example, the combination of type I metakaolin and SCW increased the rheological parameters to 56%, and the maximum compressive strength of 83.56 MPa was seen (which was found to represent an increase of 40.7% when compared with the reference geopolymer).

Metal-based composite foams are prepared by dispersed hollow particles in a metal matrix, which could improve the metal’s mechanical properties. These foams have good physical and mechanical properties and have been widely used in industrial and high-tech fields, receiving increasingly more attention by the scientific society. Changyun Li et al. successfully prepared Al_2_O_3hs_–AZ91D composites by hot-press sintering [12]. The author prepared the composite foam of the sandwich magnesium matrix by placing magnesium plates on both sides of the composite foam. They found two phenomena that occurred with the increase in the sintering temperature: (i) the actual density of the material was enhanced; (ii) the brittle MgAl_2_O_4_ phase was formed in Al_2_O_3hs_–AZ91D, and the best technological condition for preparing Al_2_O_3hs_–AZ91D was a sintering temperature of 693 K; (iii) the quasi-static and dynamic compressive strength of the Al_2_O_3hs_–AZ91D composites increased at beginning and then decreased, and the maximum values were 162MPaS and 167.87 Mpa, respectively. The new strategy of preparing metal-based composite foam with predetermined porosity that was used in this work has extensive research potential in improving the properties of lightweight and high-strength-composite-foam materials.

YbFeO_3_ is widely used as an efficient photocatalyst. Photocatalytic activity depends on its crystal structure, among which the orthogonal hexagonal structure is the best one. Due to the limitation of its metastability, nanohexagonal rare-earth orthoferrites are often irreversibly transformed into stable orthorhombic structures, which result in difficulties in synthesis. Sofia Tikhanova et al. synthesized multiphase nanocomposites containing titanium dioxide additives to stabilize h-YbFeO_3_ nanocrystals [13]. In addition, they developed new type I heterojunction nanocomposite-based o-YbFeO_3_ and hexagonal titanium dioxide and/or thorium crystals by the glycine–nitrate solution combustion method. With the addition of titanium, the mole fraction of the h-YbFeO_3_ phase in nanocomposites increased, indicating that it created a stabilizing effect by limiting the mass transfer during the heat treatment. The experimental results show that the formation of the type I heterojunction had a positive effect on the photocatalytic activity of MV in photo Fenton photodecomposition.

Rail transit train components made of lightweight aluminum alloy are one of the most important factors in the manufacture of high-performance rail transit trains, which is meaningful for reducing global environmental pollution and improving energy shortages. Therefore, Zhengwei Gu et al. evaluated the superplastic properties of 5083Al using the high-temperature tensile test and blowing forming experiments, and then fabricated the rail vehicle’s side window [14]. The result indicated that 5083Al could be obtained with the best performance in the following conditions: (i) a temperature of 510 °C; (ii) a strain rate of 5 × 10^−4^ s^−1^; (iii) a controlled maximum strain rate of 1 × 10^−3^ s^−1^; (iv) a rated pressure of 1.7 MPa. The optimum parameters of the preforming die were a depth of 14.411 mm, an area ratio of 0.378, and a friction coefficient of 0.1. Under these conditions, the average grain size increased from 21.55 µm to 28.92 µm without the phase type changing.

The performance of the cable affects its insulation, finally causing the joint to break. To solve this issue, Xuehua Wu’s group successfully changed the content of In in a Sn–1.5Cu-based solder using the brazing principle and microalloying method [15]. The authors found that the maximum conductivity of the solder reached 3.8% and 5%. In addition, the In content could reach 3.236 × 10^6^ S/m, and the highest wettability was 93.6%. Meanwhile, the minimum contact resistance of the intermediate joint was 43% lower than that of the aluminothermically welded joint. The minimum contact resistance was 7.05 µΩ when the In content was 5%, which was significantly improved compared to that of the hot-welded aluminum joint (12.39 µΩ).

The quality factor of the current solid-state thermoelectric converter is small, which means that it is obviously not enough to achieve effective thermoelectric conversion. Víctor Manuel García-Suárez designed a new thermoelectric system with physical gaps in the nanostructure [16]. According to the type of characteristics on both sides of the band gap and considering the simulation under different parameters, the highest Seebeck coefficient and quality factor could be obtained. The model proposed on this basis can be used not only to characterize and predict the thermoelectric properties of many different nanoscale junctions, but can also act as a guide for the study of other systems. These results pave the way for the design and manufacture of stable next-generation thermoelectric devices with strong characteristics and improved performance.

Nanomaterials are widely used in environmental protection and as biological antibacterial agents. SabrineZghal et al. studied the adsorption efficiency of carbon/carbon nanotube composites for the cationic dye rhodamine (RhB) in polluted wastewater to reduce the impact of synthesizing target dyes on the environment [17]. The results show that the adsorption performance of KS44-20 (containing a weight ratio of 20 wt.% of ferrocene) for dyes is better than that of KS44-0 (without ferrocene powder), because the existence of carbon nanotubes growing on the surface and in the pores produces more active sites and a greater surface area for the interaction with cationic dye RhB. In addition, the adsorption performance is related to temperature and PH, among other factors. Due to the π–π interaction between the aromatic main chain of the dye and the hexagonal skeleton of graphene as well as the carbon nanotubes, it is easy for RhB to be adsorbed. The graphite–carbon/carbon nanotube composite might play a key role in organic pollutant reduction.

AgNPs are widely developed as new bioactive materials due to their excellent antibacterial properties. However, AgNPs do not easily induce antibiotic resistance Laura Chronopoulou et al. developed a new composite of self-assembled Fmoc–Phe_3_ peptide hydrogels based on the impregnation of AgNP prepared in situ [18]. AgNPs were obtained by an in situ reduction of Ag^+^ using traditional chemical reducing agents and natural reducing agents. In addition, the material could achieve a smaller size and greater stability over time, while allowing sustainable synthesis. From the minimum inhibitory concentrations analysis, the composite showed antibacterial activity against staphylococcus aureus, which means that the hydrogel composites containing AgNPs could represent promising biomaterials for treating staphylococcus aureus-related infections.

In summary, among the ten articles published in this research topic, four papers studied the research and application of traditional nanomaterials in fields such as biomedicine, while the other six reports emphasized the excellent mechanical and mechanical properties of nanostructured materials and explored the processing modification work carried out on them. These articles indicate that nanomaterials will play a crucial role in future environmental safety and the improvement of material performance improvement. In addition, these articles pave the way for novel nanofunctional materials for their wide applications in the future.
